# F55. EFFICACY OF COMPUTER ASSISTED COGNITIVE REMEDIATION IN MID-AGED AND OLDER INPATIENTS WITH CHRONIC SCHIZOPHRENIA IN KOREA

**DOI:** 10.1093/schbul/sby017.586

**Published:** 2018-04-01

**Authors:** Kee-Hong Choi, Jinsook Kang, Sun-Min Kim, Seung-Hwan Lee, Seon-Cheol Park, Won-Hye Lee, Sun Choi, Kiho Park, Tae-Yeon Hwang

**Affiliations:** 1 Korea University; 2 Inje University College of Medicine, Ilsan Paik Hospital; 3 Inje University College of Medicine and Haeundae Paik Hospital; 4 National Center for Mental Health; 5 Yong In Mental Hospital

## Abstract

**Background:**

Accumulating evidence indicates that cognitive remediation(CR) is effective for improving various cognitive deficits in adult patients with schizophrenia. Although reports of brain plasticity in older adults and the service needs of chronic mid-aged and older patients with schizophrenia are increasing, very few randomized controlled trials of CR have been conducted in mid-aged and older inpatients with schizophrenia. We investigated the efficacy of individualized CR on the cognitive impairments of mid-aged and older inpatients with schizophrenia within the context of comprehensive psychiatric rehabilitation(PR) by comparing the results obtained with PR only and treatment as usual(TAU).

**Methods:**

Fifty-seven mid-aged and older individuals with schizophrenia (age mean: 50.07 sd: 6.01) and mild to moderate cognitive deficits were enrolled. All participants stayed in long-stay closed ward hospital. Thirty-eight who were undergoing PR were randomly assigned to CR + PR (N = 19) or PR-only (N = 19) groups. For PR groups (CR+PR group and PR only group) received comprehensive inpatient PR, including optimal pharmacotherapy, vocational rehabilitation, social skills training, daily living skills training, illness management, independent living skills training, and patient empowerment program. For CR+PR group, CR treatment was provided adjunctive to their PR program described above. CR treatment consisted of 24 sessions that occurred twice a week for 1 h/session for over 3 months. The PSSCogRehab software program which was translated in Korea and Lumosity cognitive enhancement game were utilized for CR training. Participants in PR-only group were also received same psychiatric rehabilitation program, that specific training on neurocognitive functioning was not included. Nineteen participants who were undergoing TAU without CR or PR were evaluated pre- and post-treatment.

**Results:**

No group differences were found in key demographical variables, premorbid IQ, psychiatric characteristics or baseline neurocognitive functioning at the pre-treatment. CR was easily provided and well received (drop-out rates = 5.3%) by mid-aged and older psychiatric inpatients. the CR + PR group showed greater post-treatment performance on both WCST total errors and WCST %CL compared with the PR-only group (WCST total errors: mean difference = 12.28, p = 0.026; WCST %CL: mean difference = 16.47, p = 0.017) and TAU group (WCST total errors: mean difference = 14.00, p = 0.015; WCST %CL: mean difference = 16.24, p = 0.023). However, no group differences were found on WCST total errors and WCST %CL between the PR-only and TAU groups. No group differences were found for processing speed, attention, verbal working memory or cognitive flexibility.

**Discussion:**

The results of the current study partially supported our primary hypothesis. Specifically, compared with the PR-only and TAU groups, the CR + PR group showed greater improvement in executive functioning. Importantly, the reliable change index(RCI) indicated that more participants in the CR + PR group had clinically meaningful improvements in LM and executive functioning compared with participants in the PR-only and TAU groups. These results suggested that CR improved some cognitive deficits in mid-aged and older long-stay inpatients with schizophrenia and that it was effective as an adjunctive treatment to the usual PR services provided in inpatient settings.

